# An Innovative IoT and Edge Intelligence Framework for Monitoring Elderly People Using Anomaly Detection on Data from Non-Wearable Sensors

**DOI:** 10.3390/s25061735

**Published:** 2025-03-11

**Authors:** Amir Ali, Teodoro Montanaro, Ilaria Sergi, Simone Carrisi, Daniele Galli, Cosimo Distante, Luigi Patrono

**Affiliations:** 1 Department of Engineering for Innovation, Università del Salento, 73100 Lecce, Italy; amir.ali@unisalento.it (A.A.); teodoro.montanaro@unisalento.it (T.M.); ilaria.sergi@unisalento.it (I.S.); cosimo.distante@cnr.it (C.D.); 2 Gematica s.r.l., 73100 Lecce, Italy; s.carrisi@gematica.com (S.C.); d.galli@gematica.com (D.G.); 3 Institute of Applied Sciences and Intelligent Systems of CNR, 73100 Lecce, Italy

**Keywords:** IoT, healthcare, elderly, edge computing, anomaly detection, real-time health monitoring, non-wearable

## Abstract

The aging global population requires innovative remote monitoring systems to assist doctors and caregivers in assessing the health of elderly patients. Doctors often lack access to continuous behavioral data, making it difficult to detect deviations from normal patterns when elderly patients arrive for a consultation. Without historical insights into common behaviors and potential anomalies detected with unobtrusive techniques (e.g., non-wearable devices), timely and informed medical interventions become challenging. To address this, we propose an edge-based Internet of Things (IoT) framework that enables real-time monitoring and anomaly detection using non-wearable sensors to assist doctors and caregivers in assessing the health of elderly patients. By processing data locally, the system minimizes privacy concerns and ensures immediate data availability, allowing healthcare professionals to detect unusual behavioral patterns early. The system employs advanced machine learning (ML) models to identify deviations that may indicate potential health risks. A prototype of our system has been developed to test its feasibility and demonstrate, through the application of two of the most frequently used ML models, i.e., isolation forest and Long Short-Term Memory (LSTM) networks, that it can provide scalability, efficiency, and reliability in the context of elderly care. Further, the provided dashboard enables caregivers and healthcare professionals to access real-time alerts and longitudinal trends, facilitating proactive interventions. The proposed approach improves healthcare responsiveness by providing instant insights into patient behavior, facilitating more accurate diagnoses and interventions. This study lays the groundwork for future advancements in the field and offers valuable insights for the research community to harness the full potential of combining edge computing, artificial intelligence (AI), and the IoT in elderly care.

## 1. Introduction

As the global population continues to age, with projections indicating that the number of people aged 65 and over will nearly double by 2050 [1], the need for innovative elderly care solutions is becoming increasingly important. Ensuring the safety and well-being of elderly individuals living independently is paramount, as this demographic shift poses significant challenges to healthcare systems worldwide [2]. Effective monitoring systems are crucial for detecting early signs of potential health issues and enabling timely interventions to prevent serious incidents, thereby reducing the burden on healthcare resources. In this context, the absence of continuous behavioral data significantly impedes the ability of doctors to detect deviations from normal patterns when elderly patients arrive for a consultation, making informed medical interventions challenging [3].

However, existing monitoring systems, predominantly relying on cloud computing for data analysis and storage [4,5], face significant limitations. These systems offer scalability but introduce challenges such as latency in response times, which can be detrimental in situations where immediate intervention is necessary [6]. Furthermore, privacy concerns are significantly heightened with cloud computing [7], as the transmission and storage of sensitive health data offsite pose risks of unauthorized access and breaches. Moreover, the current landscape of elderly monitoring solutions often incorporates wearable devices to track health metrics and activity levels. Despite their potential, these devices frequently encounter barriers related to user compliance and comfort. Elderly individuals may neglect to wear such devices consistently due to discomfort or forgetfulness, leading to gaps in monitoring and data collection, which undermine the reliability of health assessments [8].

Addressing these multifaceted challenges, this paper presents an Internet of Things (IoT) system that utilizes a combination of the IoT, AI, and edge computing techniques for monitoring and supporting elderly adults and their caregivers in smart home environments. Unlike traditional approaches, our system leverages non-wearable sensors integrated throughout the home environment to ensure unobtrusive surveillance without requiring any active effort from elderly individuals, thereby circumventing the compliance issues associated with wearables. We focus on developing an edge-based framework that processes data locally within the smart home environment. This approach enhances data privacy and security and minimizes latency, allowing for immediate analysis and response. A prototype of our system has been developed to test its feasibility and demonstrate that it can provide scalability, efficiency, and reliability in the context of elderly care. The developed prototype utilizes existing datasets to experiment with how anomaly detection techniques can help doctors in their daily routines through a dashboard that displays the collected data and predictions. By employing two of the most frequently used ML models for anomaly detection, i.e., isolation forest and LSTM networks [9,10], our experiments effectively identify behavioral deviations that may indicate potential health risks.

The selection of the two models was guided by their characteristics: the isolation forest algorithm is an advanced ML technique known for its effectiveness in identifying outliers within intricate, multidimensional datasets. It excels at detecting subtle yet critical anomalies that conventional techniques frequently miss. Additionally, we incorporate an LSTM network, which is known for its ability to recognize temporal dependencies in sequential data. By analyzing patterns over time, LSTM networks enable the prediction of health issues based on identified anomalies. Moreover, a real-time monitoring dashboard accessible to caregivers and medical professionals provides immediate alerts and comprehensive updates about the patient’s status. This dashboard facilitates prompt and informed decision-making, improving response times and ultimately enhancing the quality of care for elderly people.

This work provides the foundation for future developments in the field and provides the research community with a valuable basis for fully leveraging the advantages of combining edge computing, AI, the IoT, and ML.

The main contributions of this work are as follows:

- We propose a non-wearable sensor-based IoT system in smart homes, eliminating the need for wearable devices and ensuring continuous and uninterrupted data collection.
- We design an edge-centric architecture that processes data locally within smart home environments, reducing latency and enhancing security while safeguarding patient privacy.
- We introduce an advanced anomaly detection approach for temporal analysis to identify subtle health risk indicators before they become critical issues.
- We engineer a real-time monitoring dashboard that provides immediate alerts and detailed health status updates, facilitating rapid and informed decision-making.
- We demonstrate the scalability and reliability of a functional prototype in real-world elderly care scenarios through extensive testing performed using isolation forest for spatial analysis and LSTM networks applied to existing datasets.

The remainder of this paper is organized as follows. Section 2 analyzes related works, while Section 3 presents the proposed architecture. Section 4 discusses the implementation details that could guide future or similar works. Section 5 reports the details of the experiments, and Section 6 discusses the results obtained from validating the proposed system. Finally, Section 7 concludes this paper and discusses future work.

## 2. Related Works

The field of smart home technology for elderly care has advanced considerably in recent decades, especially in systems that monitor daily activities to identify health risks. Traditional methods often rely on wearable devices to collect data and cloud AI-based solutions for their processing. Such solutions usually face challenges related to data privacy, due to the risks of sharing data over the internet; latency, due to the time needed to transfer data to the cloud and wait for results; and user compliance issues, due to the unwillingness of elderly individuals to wear devices. In response, recent innovations in non-wearable sensor networks integrated with edge computing frameworks offer enhanced privacy and real-time data processing. However, many of these systems do not focus on utilizing anomaly detection techniques applied to non-wearable data for health monitoring, which is the main focus of our research. Various studies have already been proposed in the literature that utilize ML models to process non-wearable data acquired in the home environment for health monitoring.

For instance, in the study by Paudel et al. [11], the authors explored anomaly detection to monitor the activities of elderly individuals in smart homes using a graph-based approach. They utilized the Kyoto dataset from the Center for Advanced Studies in Adaptive Systems (CASAS) [12] program, mapping daily routines via non-wearable sensors like motion, temperature, and door sensors. By examining activation frequency, duration, and spatial patterns, they identified deviations from normal behavior. Despite providing detailed insights, the study faced limitations, including cloud processing burdens, privacy concerns, and scalability issues due to complex graph structures and extensive sensor data. Our approach tackles these challenges by using lightweight algorithms like isolation forest for efficient anomaly detection without complex graphs, thereby enhancing system performance and scalability. In addition, through the utilization of edge computing, our work addresses the privacy and latency issues experienced by other authors.

Another interesting study was conducted by Shahid et al. [13]. It explored the use of anomaly detection algorithms in elderly care using non-wearable IoT devices in single-resident apartments. The study tracked kitchen and bathroom visits to establish behavioral norms, detect anomalies from deviations, and notify caregivers via SMS. Despite its successes, cloud processing delays hindered real-time analysis, and reliance on long-term norms reduced responsiveness to sudden activity changes. In contrast, our approach enables the use of advanced ML techniques, such as the isolation forest and LSTM models, to enhance adaptability and enable real-time detection of both static outliers and dynamic changes. Furthermore, using edge computing ensures responsive, privacy-preserving elderly monitoring and health risk detection. Gupta et al. [14] employed an LSTM-based neural network for fall detection in smart homes. However, its narrow focus limited health monitoring, and its reliance on cloud-based infrastructure introduced latency. Our work extends beyond fall detection by integrating sensors for sleep, motor function, and social interaction (i.e., periods of reduced engagement and low interaction with others that may signal a need for intervention), which are important indicators of cognitive health. Edge computing ensures local processing, thereby reducing latency and enhancing privacy. This enables real-time anomaly detection for comprehensive and prompt elderly care, unlike Gupta’s fall-focused model, offering an expanded perspective on elderly monitoring.

Novak et al. [15] proposed an anomaly detection approach for elderly care in smart homes using non-wearable sensors like PIR and pressure sensors. Their methodology combines Self-Organizing Maps (SOMs), an unsupervised learning method for clustering and visualization on the cloud [16], and Markov models [17] to predict subsequent activities. By analyzing activities, location, timing, and duration, it establishes normal behavior and identifies deviations, which are crucial for monitoring the well-being of elderly residents. However, while the approach leverages the non-intrusive nature of sensors, it faces challenges in real-time anomaly detection and has limitations in the dataset’s applicability for elderly monitoring. In comparison, our system is designed to utilize various ML algorithms on the edge and collect data from various devices in real time.

Pérez-Toro et al. [18] advanced Alzheimer’s assessment using ForestNet, a hybrid ML framework combining decision trees and neural networks to analyze acoustic features. It achieved high recall in patient classification while identifying key diagnostic markers like rhythm and voice rates. Its interpretability provides clinicians with clear and actionable insights, bridging the gap between complex ML models and clinical practice. The study faced limitations, such as balancing interpretability with high predictive accuracy. However, although the study aimed to ensure privacy when sending data to the cloud, it did not utilize an edge system. Therefore, the system proposed in this paper could enhance reliability through edge computing and offer greater flexibility by enabling the integration of additional sensors and devices for real-time data collection.

Susnea et al. [19] used non-wearable sensors, such as Passive Infrared (PIR) motion detectors and magnetic door contacts, to monitor the activities of elderly individuals living alone. These sensors are integrated into a smart home environment to create activity maps that reflect spatial distribution and intensity. Their method uses encoded sensors to detect deviations from behavioral routines tested on the CASAS, HH126, and Kasteren House C datasets. The approach highlights the system’s low cost, scalability, adaptability, minimal expert knowledge required for setup, and robust privacy protection; however, it is limited by the need for cloud infrastructure to process the data. Our system enhances this approach by leveraging edge computing and integrating additional sensors and devices to collect real-time data. Furthermore, it tests different ML techniques and incorporates previously unreported performance evaluations.

Sadhu et al. [20] demonstrated the potential of using ML applications on edge and fog computing architectures for remote assessment of Parkinson’s disease. Their study uses smart gloves with flex sensors and an inertial measurement unit to capture hand movements, which are processed by ML classifiers for accurate, low-latency Parkinsonian movement classification. The edge–fog approach effectively addresses the latency and privacy concerns often associated with cloud-based telehealth systems, marking a significant advancement in real-time motor symptom monitoring. However, their approach remains limited to Parkinsonian symptom tracking, lacking a broader focus on cognitive health assessment. In addition, the use of an intrusive wearable device limits the effectiveness of the proposed solution.

Furthermore, Mokhtari et al. [21] proposed a hierarchical federated learning-based anomaly detection method for smart healthcare. The integration of edge computing with wearable and medical devices was explored to enhance data privacy and reduce latency in healthcare applications. While their research highlighted the advantages of using edge computing for anomaly detection, it was limited to the use of wearable devices. Our work extends these concepts specifically to non-wearable sensors within smart home environments, focusing on elderly care. The publication inspired our work, particularly in utilizing edge-based processing, enabling the efficient and secure detection of potential health anomalies and meeting the growing need for decentralized healthcare monitoring solutions.

Another interesting study that focused on the application of edge computing for human activity recognition was conducted by Singh et al. [22]. Their work presented a Human Activity Recognition (HAR) system employing a Convolutional Neural Network (CNN) designed for edge devices. Their approach employs wearable sensors, which efficiently process sensor data locally on edge devices, significantly reducing latency and enhancing data privacy. However, the utilization of wearable devices limited the effectiveness of the performed estimations.

Another notable study was conducted by Wang et al. [23]. After conducting a comprehensive analysis of the state of the art, demonstrating the need for frameworks to facilitate the application of ML, the IoT, and edge computing to the healthcare sector, the authors proposed an edge-based framework to process data from wearable sensors, ensuring timely detection of health anomalies and reducing latency in critical healthcare applications. The lack of support for non-wearable devices and the exclusive use of deep learning techniques for ML analysis limits the applicability of their framework to scenarios involving only wearable devices. However, it motivated us to incorporate support for non-wearable devices and apply lightweight anomaly detection techniques directly on the edge to detect anomalous behaviors in elderly patients.

The study conducted by Rancea et al. [24] further motivated our work. The authors presented an overview of the current landscape of edge computing intersecting with the healthcare domain, highlighting its role in providing innovative solutions in the context of the IoT revolution. The study discussed the critical role that edge-based systems play in the healthcare sector and underscored the need for real-time data processing and rapid decision-making in AI-optimized edge computing-based healthcare systems. In addition, it gave an overview of the advantages that solutions similar to the one proposed in this paper can offer in monitoring patients and providing prompt notifications to provide doctors with the necessary information for making well-informed decisions.

Additionally, Liu et al. [25] acknowledged the broader landscape of edge computing in healthcare by demonstrating significant improvements in energy efficiency within edge computing frameworks. Their work pursued a green computing design and formulated an optimization problem that minimizes the long-term energy consumption of the proposed network, reduces computing delays, and minimizes data requirements. A combination of their solution with the one proposed in this paper could significantly reduce computing delays and data requirements while preserving privacy and related characteristics that we demonstrated to be highly important in the healthcare domain.

Finally, it is worth mentioning the review presented by Punugoti et al. [26], who explored healthcare IoT and edge computing’s benefits and drawbacks. Through a literature review, the authors demonstrated that edge computing can improve healthcare delivery, costs, and outcomes. In addition, they evaluated edge computing’s enabling technologies, applications, and obstacles in healthcare and discussed possible solutions. As highlighted by the authors, existing research lacks solid empirical evaluations of proposed solutions. Furthermore, the proposed solutions do not seem ready to integrate real-time monitoring and ML techniques for data from non-wearable devices, as demonstrated in this paper. Therefore, the review further motivated our work and informed our suggestions for promising directions for future work, which will be discussed in the conclusions.

## 3. System Architecture

This research introduces a novel edge-based Internet of Things (IoT) framework that enables real-time monitoring and anomaly detection using non-wearable sensors to assist doctors and caregivers in assessing the health of elderly patients. Specifically, it leverages edge computing supported by cloud computing to locally process data using anomaly detection techniques and provide innovative services (e.g., a dashboard for doctors and an application for caregivers).

**Figure 1:**
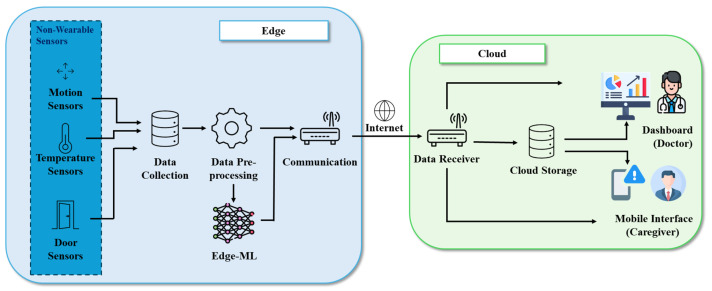
System architecture.

The envisioned system architecture, as shown in Figure 1, is divided into two main blocks: an edge block and a cloud block, each tailored to specific functionalities within the healthcare monitoring system. The edge block handles local data acquisition, preprocessing, and analysis, ensuring minimal latency and data privacy. Meanwhile, the cloud block provides long-term storage, trend analysis, and user interfaces for stakeholders such as doctors and caregivers. This modular and scalable architecture is designed to adapt to evolving technologies and growing requirements in elderly care. By leveraging local processing to reduce latency and cloud computing for storage and analytics, the system ensures timely, secure, and effective monitoring. The following subsections detail the components and roles of each block.

### 3.1. Edge Block

The edge block is the cornerstone of real-time operations in the system. It processes data locally, enabling swift anomaly detection and immediate responses while ensuring data privacy by reducing the need to transmit raw data over external networks. This modular block allows seamless integration of additional sensors, algorithms, or components without significant architectural changes. Therefore, the edge block is responsible for all operations that must be performed on the raw data, including processing. Local processing of data can guarantee precise predictions (which may only be possible with the collected raw data) and personalization, which are necessary for contacting the cloud block to display alerts or inferences to doctors and caregivers. This block consists of various modules: “non-wearable sensors” (such as motion sensors, temperature sensors, and door sensors), “data collection”, “data preprocessing”, “edge-ML”, and “communication” modules, all operating at the edge of the network.

#### 3.1.1. Non-Wearable Sensors

A home environment is typically equipped with various non-wearable sensors, including environmental sensors, location tracking systems, temperature monitors, motion detectors, and activity monitoring systems. These sensors continuously collect data on daily activities and health parameters. Our architecture leverages these sensors to collect environmental data, enabling continuous, non-intrusive monitoring tailored for real-time health and activity analysis. The use of environmental sensors provides a reliable and unobtrusive alternative to wearable devices, eliminating the dependency on user compliance. Wearables often require charging, regular use, and proper maintenance, which may not be practical for elderly individuals or those with physical or cognitive limitations. By focusing on environmental data, our system ensures continuous monitoring while reducing the burden on the user. Environmental sensors capture data passively and continuously, offering a broader perspective compared to wearable devices. While wearables primarily provide individual metrics like heart rate or step count, environmental sensors monitor the entire living environment. For instance, a motion sensor can detect whether an individual has not moved from one room to another for an unusually long time, which may indicate a potential problem. Similarly, door sensors can track exits to ensure safety, particularly for individuals with dementia who may wander. Additionally, temperature sensors can identify unsafe environmental conditions, such as a dangerously cold room. These capabilities make non-wearable sensors a holistic and practical solution for comprehensive monitoring, especially in sensitive applications like elderly care. The flexibility of our architecture lies in its modular design, which allows seamless integration of additional devices as they become available, whether real or simulated. Real devices installed in a home can send live data directly to the system, while simulated devices can be utilized during testing or in scenarios where physical installation is not feasible. This adaptability ensures that the system remains scalable, future-proof, and capable of incorporating emerging technologies.

#### 3.1.2. Data Collection

The data collected by the sensors are first aggregated through the data collection module, which ensures that all relevant information is captured and prepared for further processing. This module acts as the initial interface between the sensors and the rest of the system, gathering data from various sources such as motion, temperature, door, and environmental sensors. To support future adaptability, the data collection module is designed as a stand-alone sub-framework capable of integrating new bundles at runtime (e.g., by integrating and adapting existing frameworks such as the Domotic OSGi Framework (DOG) [27] as an external module or by developing an innovative internal module) to handle a variety of communication protocols (such as MQTT, HTTP, or CoAP) and data formats (such as JSON, XML, or binary formats). Its intrinsic modularity ensures compatibility with both existing and new devices. This capability allows it to seamlessly integrate with sensors using different standards and technologies. The module also performs preprocessing tasks, including filtering noise, standardizing data formats, and applying basic validation to ensure that only accurate and meaningful data are forwarded to the next stage. By supporting multiple protocols and formats, the data collection module is a versatile and future-proof component, enabling the system to scale and adapt to emerging technologies and new devices as they become available.

#### 3.1.3. Data Preprocessing

The data preprocessing module is a critical component designed to preprocess incoming sensor data in real time, ensuring it is structured and ready for further analysis. This module is responsible for filtering out noise, normalizing data from multiple sources, and extracting essential features to facilitate accurate anomaly detection. Its primary role is to transform raw, unprocessed data into a clean and interpretable format for subsequent machine learning analysis. This module is designed to handle high volumes of data efficiently, supporting the real-time nature of the system. It is scalable to accommodate additional sensors and adaptable to evolving requirements, ensuring compatibility with diverse data formats and structures. By optimizing data quality and reducing computational complexity, the data preprocessing module ensures that downstream modules, such as the “edge-ML” and the modules in the cloud, can operate effectively. The advantages of this module include its high throughput, reliability, and ability to process data without introducing latency. These characteristics make it a foundational part of the architecture, supporting the goal of the system of real-time, unobtrusive elderly care monitoring by enabling seamless integration of environmental sensor data, rapid preprocessing, and continuous anomaly detection.

#### 3.1.4. Edge-ML

The edge-ML module is designed to analyze preprocessed data to detect patterns and deviations from normal behavior. Its primary role is to identify potential anomalies in the data, which may indicate health risks or unusual activity patterns in the monitored environment. By processing data outputs from the data preprocessing module, the edge-ML module evaluates the resident’s behavioral trends and flags irregularities for further action. In addition to detecting anomalies, it generates real-time notifications based on identified irregularities or critical events. These notifications are routed through the communication module to the cloud infrastructure and are displayed on the monitoring “dashboard” and “mobile interface”, ensuring that caregivers and healthcare providers are promptly informed of potential issues. This integration enables timely alerts and actionable insights to be delivered to caregivers and healthcare providers, facilitating prompt intervention when necessary. The design of this module ensures that it is adaptable to various types of data and capable of handling complex patterns, supporting the system’s overall goal and enhancing decision-making for elderly care.

#### 3.1.5. Communication

The communication module facilitates the flow of data between local processing units and external systems in the cloud block. It ensures that only relevant and processed data are securely transmitted to the cloud storage, reducing the exposure of sensitive raw data over external networks. In addition to data transmission, the communication module is responsible for routing real-time notifications generated by the edge-ML module to the dashboard and the mobile interface. This guarantees that caregivers and healthcare providers are promptly informed of detected anomalies or critical health events, enabling timely intervention. By efficiently managing both data and notifications, the communication module acts as a critical hub for real-time and secure communication within the system.

### 3.2. Cloud Block

The cloud block is responsible for providing long-term storage, trend analysis, and user interfaces for stakeholders such as doctors and caregivers. It is responsible for all operations that do not require access to the raw data. The cloud block displays alerts and inferred information communicated by the edge block after the processing of the collected raw data. This block consists of different modules: “data receiver”, “cloud storage”, “dashboard”, and “mobile interface” modules, all operating in the cloud.

#### 3.2.1. Data Receiver

The data receiver is the first module of the cloud block and acts as the gateway for data transitioning from the edge to the cloud. It ensures the secure, efficient, and reliable reception of data streams transmitted over the network. Upon receiving the data, the data receiver performs integrity and validation checks to verify that the data have not been corrupted or altered during transmission. These checks are essential for maintaining the reliability and trustworthiness of the data, which serve as the foundation for subsequent analyses and healthcare interventions. Beyond validation, the data receiver is responsible for sorting and filtering incoming data based on predefined parameters, such as data type, source, and priority. This organization optimizes the data management pipeline and ensures that only relevant and actionable information is processed or stored. The data receiver operates within an event-driven architecture, enabling the distribution of data to various cloud-based services and applications dynamically. This dynamic distribution is crucial for triggering immediate responses, such as sending alerts to caregivers or updating the dashboard in real time. By supporting a service-oriented approach, the data receiver enables different components within the cloud infrastructure to subscribe to specific data streams. This ensures the efficient dissemination of actionable insights to all relevant stakeholders, transforming raw data into meaningful outputs that drive healthcare monitoring and interventions.

#### 3.2.2. Cloud Storage

The cloud storage is designed to store processed data for long-term archival and analysis. Its primary role is to maintain an organized repository of health and activity data for trend analysis and historical reviews. Unlike the real-time operations occurring on the edge, cloud storage supports non-time-sensitive functions, such as generating insights from long-term data and supporting data visualization tools. The data stored in this module can be accessed by the dashboard, where caregivers and healthcare providers review trends and patterns to make data-driven decisions. By offloading archival functions to the cloud, the system ensures scalability and supports future expansion to accommodate increasing data volumes.

#### 3.2.3. Dashboard (Doctor) and Mobile Interface (Caregiver)

The dashboard and the mobile interface work together to provide comprehensive and accessible monitoring for both healthcare providers and caregivers. The dashboard, designed for doctors, offers a detailed and data-rich platform to track the resident’s health and activity patterns in real time. It displays anomaly alerts generated by the edge-ML module, such as irregular activity patterns, prolonged inactivity, or unsafe environmental conditions, along with contextual details like timing and severity. In addition to these alerts, the dashboard presents health status metrics to monitor cognitive decline over time. For the experiments described in the next section, these metrics include motor function, evaluated using sitting-to-walking ratios; sleep quality, analyzed through wake-up patterns and disturbances; and social interactions, assessed based on home occupancy trends and room transitions, with periods of reduced engagement and low interactions with others potentially signaling a need for intervention. The dashboard’s ability to visualize historical data allows healthcare providers to identify trends, evaluate long-term cognitive changes, and adjust care plans proactively. The mobile interface, on the other hand, is tailored for caregivers and focuses on simplicity and immediacy. It delivers real-time notifications about detected anomalies, such as inactivity, unusual motion, or hazardous environmental conditions, ensuring that caregivers are promptly informed and able to respond quickly. While it also provides health status metrics, these are presented in a more simplified format compared to the dashboard. In our scenario, as described in the next sections, caregivers can check the current sitting-to-walking ratio for mobility insights, recent sleep disturbances for assessing rest patterns, and updates on home occupancy trends for understanding social interaction. Designed for on-the-go access, the mobile app allows caregivers to stay connected and informed regardless of their location, prioritizing quick actions and immediate interventions.

## 4. Implementation

This section provides a comprehensive exploration of the methodologies used to develop a cutting-edge IoT system implementing the defined system architecture. This includes details of the various modules in the edge and cloud blocks, such as the communication, data preprocessing, edge-ML, and cloud storage blocks, and their integration with the dashboard and the mobile interface.

To validate the feasibility of advanced analytics within the designed edge computing framework, we decided to concentrate most of the effort in this research paper on only some of the architecture blocks. Specifically, we concentrated on (a) the modules responsible for the ML algorithms and models utilized at the edge, such as the data preprocessing module and the edge-ML module, and (b) the cloud block implementing cloud storage and the dashboard. Therefore, we decided to utilize existing datasets to simulate the presence of real-time sensors that send data to the system while leaving the implementation of other blocks (e.g., mobile interface) for future work.

This preliminary phase is crucial for assessing the practicality of real-time anomaly detection and serves as a foundational step toward realizing the full potential of the proposed architecture. To ensure scalability, the system is designed to allow easy addition of more computational nodes at the edge.

### 4.1. Data Collection and Preprocessing

The data collection and preprocessing modules ensure that data are prepared for effective anomaly detection and ML applications. To simulate real-time sensor data, the TM029 dataset from CASAS [12] was chosen. This dataset comprehensively represents daily activities in a single-resident smart home environment, making it ideal for analyzing behavioral patterns and detecting anomalies. This dataset includes readings from the following sensors:

- Motion Sensors (ZigBee Round Motion Detectors WMS10-C-ZP [28]): These sensors capture activity levels and mobility, which are essential for understanding movement patterns and energy expenditure.
- Door Sensors (ZigBee Magnetic Contact/Temperature Sensor WCS10A-2-ZP [28]): These sensors monitor room transitions and occupancy, providing insights into room usage and time spent in different areas of the home.
- Temperature Sensors (ZigBee Magnetic Contact/Temperature Sensor WCS10A-2-ZP [28]): These sensors add contextual information about environmental conditions that may impact comfort, health, and overall activity.

The raw sensor data required extensive preprocessing to ensure that it was suitable for analysis and model training. Missing values in the dataset were addressed using forward-filling techniques [29], a method that maintained temporal continuity and prevented disruptions in downstream analysis, which is especially critical for time-series data. Forward-filling replaces missing data points with the most recent valid observation, ensuring that the integrity of time-series patterns is preserved. Several key features were derived from the preprocessed data to capture meaningful activity and behavioral trends. These included sitting duration, representing the time spent seated during the day; walking duration, which measured the total time spent moving within the home; wake-up count, capturing the frequency of awakenings during sleep periods; out-of-home duration, reflecting the time spent outside the home; and sleep duration, indicating total sleep time as a measure of rest quality and overall health. Table 1 summarizes these parameters and their derivations.

These features were carefully selected for their relevance in identifying behavioral anomalies and potential health risks. To ensure consistency across all features, min-max scaling was applied [30]. This normalization technique transforms data into a range suitable for ML models, ensuring uniformity and comparability between features. The preprocessing tasks were implemented using Python [31], leveraging well-established libraries to ensure efficiency and accuracy. The “pandas” library [32] was utilized for efficient data manipulation and preprocessing, providing robust capabilities for handling structured datasets. Additionally, the NumPy library [33] was employed for numerical computations and array operations, enabling seamless transformations and calculations during the preprocessing phase. These tools ensured that the raw sensor data were efficiently transformed into a structured format suitable for analysis and ML applications.

### 4.2. Technology Integration and System Configuration

The core infrastructure of our IoT system is built upon a carefully selected technology stack, comprising Node.js [34,35] and MySQL [36,37], chosen for their proven reliability and suitability for real-time applications in elderly care monitoring. This combination ensures efficient data handling, robust storage, and scalability, critical for the system’s performance in real-world conditions. Node.js [34] is at the heart of our data processing operations due to its highly efficient, non-blocking, event-driven architecture. This technology is particularly well suited for applications that require real-time capabilities, as it can handle numerous data streams concurrently without delays. In our system, Node.js [34] is a central component of the data processing layer, orchestrating data flow from various sensors scattered throughout the care environment. Positioned within our IoT infrastructure, Node.js [34] manages and processes numerous data streams concurrently without delays, ensuring that all data are processed swiftly and accurately. This rapid processing capability is crucial for the timely detection of anomalies and potential emergencies, enabling immediate response and intervention. Node.js [34] also facilitates the development of scalable network applications. Its lightweight runtime supports our system’s requirement for high throughput and data-intensive handling, which are essential for monitoring the nuanced behaviors of elderly residents. Node.js [34] also facilitates the integration of real-time data processing with other system components, such as the database management system (MySQL [37]) and the user interface, thereby enhancing the overall responsiveness and efficiency of our elderly care monitoring solution. Furthermore, Node.js [34] integrates seamlessly with Express.js [38], which serves as the framework for incoming and outgoing data. This setup manages data flow from various sensors and data processing units, ensuring a smooth and scalable communication pipeline. Complementing Node.js [34], MySQL [37] provides robust data management capabilities. Chosen for its reliability and efficiency, MySQL stores and manages the vast quantities of data generated by the IoT sensors. It ensures efficient data retrieval with features like indexing, which speeds up the search process, and query optimization, reducing the time to execute database commands. MySQL handles structured data storage with high transactional requirements in our deployment, ensuring data integrity and security. The advanced features of MySQL, such as partitioning, are utilized to manage large tables and complex queries more efficiently. This functionality is particularly important given the continuous data influx from our monitoring devices. The modular nature of our system allows the easy integration or substitution of the MySQL database with other similar solutions, like NoSQL databases. However, the choice was guided by the requirements of our experiment: While semi-structured data from sensors could potentially be managed using no-SQL databases, we opted for MySQL due to its robust transactional support, which is crucial for ensuring data integrity and consistency in our anomaly detection processes. Additionally, MySQL’s advanced querying capabilities allow for the precise and complex data manipulations necessary for the sophisticated analysis models our system employs, which are integral to accurately processing structured sensor data streams.

In addition, to ensure seamless communication between the sensors and the data processing unit, the system employs MQTT (Message Queuing Telemetry Transport), one of the most frequently used publish/subscribe protocols for the IoT [39]. MQTT was chosen for its widespread adoption in healthcare IoT solutions, where its lightweight protocol facilitates efficient real-time communication, which is crucial for the rapid transmission of sensor data to the system. This capability allows the system to detect and process anomalies, ensuring timely intervention. The choice of MQTT over alternatives such as CoAP was guided by its documented advantages in similar applications across the healthcare sector, providing the necessary reliability and efficiency for our system’s needs [40].

The integration of Node.js [34], Express.js [38], MySQL [37], and MQTT ensures that the system operates efficiently, handling the demands of real-time data processing, secure storage, and high-speed communication. This technological foundation is designed to scale with future enhancements, such as adding more sensors or supporting additional users, ensuring that the system remains robust and reliable under varying operational loads.

### 4.3. Edge-ML Module: Machine Learning Models and Anomaly Detection Techniques

The anomaly detection component utilizes two machine learning algorithms: isolation forest [41] and Long Short-Term Memory (LSTM) [14]. These models were chosen among the most frequently used anomaly detection algorithms for their ability to complement each other in detecting both isolated anomalies and time-dependent patterns in behavioral data. Isolation forest was selected for its ability to effectively identify anomalies in high-dimensional data. The algorithm works by isolating data points through random partitioning, where anomalies are easier to isolate due to their distinct characteristics. Isolation forest is well suited for detecting outliers in datasets with multiple features, such as those related to motion, door, and temperature sensor data. It was chosen because of its efficiency in handling large datasets and its ability to detect anomalies without relying on prior knowledge of the data distribution, making it ideal for real-time applications in a smart home environment. The isolation forest model was configured with 100 trees (n_estimators = 100), using a contamination factor of 0.1, and its training was executed efficiently within a few seconds (an average of 4 s).

In addition, Long Short-Term Memory (LSTM) networks were employed to capture temporal dependencies in the data. LSTM networks are specifically designed to work with sequential data, which is crucial for identifying anomalies over time. Recent studies, such as [42], have demonstrated that LSTM-based models outperform traditional statistical methods in anomaly detection, particularly in healthcare applications, due to their ability to effectively model both short-term and long-term dependencies. In our case, behavioral patterns, such as changes in walking duration, sitting duration, or wake-up count, are best understood in a temporal context. The LSTM model was chosen because it excels at modeling time-series data, making it well suited for predicting future behavioral states and detecting deviations from expected patterns. The LSTM model was configured with two layers, each with 50 units, and was trained over 20 epochs with a batch size of 32. An early stopping mechanism was employed after five epochs without improvement to prevent overfitting. The entire training process was optimized to be completed in a few minutes (an average of 8 min).

Both models were trained on an 80/20 train-test split of the dataset, which means that 80% of the data were used for training the models and the remaining 20% for testing, ensuring robust evaluation. Early stopping (a technique used during training) was employed to prevent overfitting. This method monitors the model’s performance on a validation set during training and stops the training process once the model performance ceases to improve, thus ensuring that the model generalizes well to new, unseen data. Input data for both models were normalized using min-max scaling to ensure consistency across features. Min-max scaling is a preprocessing technique that transforms the features into a fixed range, typically 0 to 1, by subtracting the minimum value of each feature and then dividing by its range, thereby enhancing predictive accuracy.

The isolation forest model is expected to effectively flag irregularities in individual data points, such as sudden changes in sitting or walking durations. Meanwhile, the LSTM network was designed to predict future activity sequences, with anomalies flagged when the predictions deviate significantly from actual behavior. This configuration allows the system to identify both isolated incidents of anomalous behavior and broader shifts in activity patterns that may indicate cognitive or physical decline.

**Figure 2:**
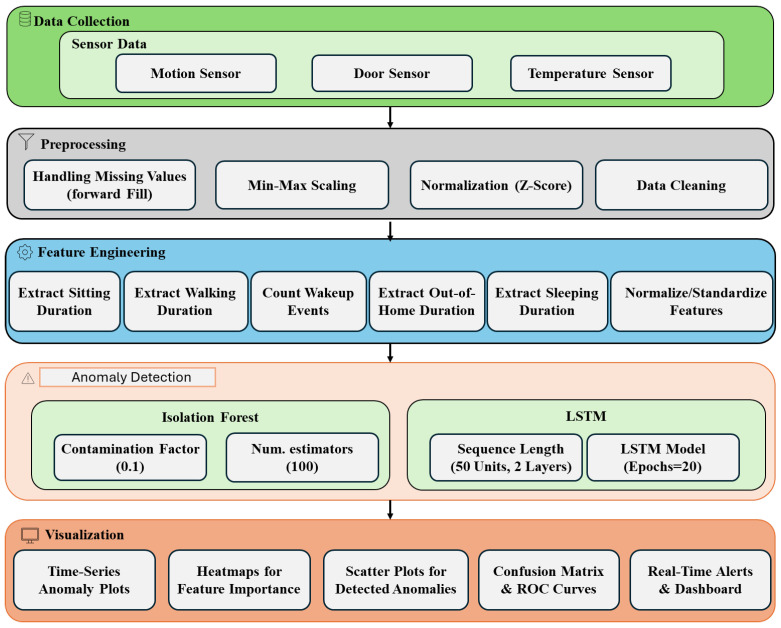
Data flow diagram illustrating the steps involved in anomaly detection, including sensor data collection, data preprocessing, feature engineering, machine learning, and visualization.

To enhance the understanding of the anomaly detection pipeline, Figure 2 illustrates the data flow from sensor collection to preprocessing, feature engineering, machine learning, and visualization. The diagram provides a concise visual representation of how the isolation forest and LSTM models are integrated within the system, offering a clear view of the relationships and processing steps. As already stated, in our experiments, we used the CASAS dataset, in which data collection involved continuous monitoring through non-wearable sensors deployed in a smart home environment. These sensors gathered information on motion activity, room occupancy, temperature variations, and door usage, providing a rich dataset for behavioral pattern analysis. The preprocessing stage ensures data quality and consistency by applying noise filtering, missing value interpolation, and min-max normalization, which standardizes feature values for uniform input to the models. Following preprocessing, feature engineering extracts key metrics that aid in anomaly detection. This includes statistical features, such as mean, variance, and standard deviation; temporal features, such as activity duration and frequency of movement; and event-based features that track sensor activations over time. These engineered features serve as inputs to the anomaly detection models, each employing a distinct approach to identifying irregularities. The isolation forest model assigns an anomaly score to each data point based on how easily it can be isolated, effectively detecting sudden behavioral deviations like prolonged inactivity or unusually frequent movements. In contrast, the LSTM model leverages time-series predictions, learning behavioral patterns from historical data and identifying anomalies when actual behaviors significantly deviate from predicted sequences. The final phase of the pipeline is visualization, where the detected anomalies are represented through time-series plots, heatmaps, and behavioral trend graphs. These visual outputs enable medical professionals and caregivers to interpret changes in behavior over time, allowing for early intervention in case of potential health risks.

### 4.4. Dashboard Development and Integration

Our system features an interactive dashboard developed using React.js [43] to provide caregivers and doctors with real-time insights into the health and activities of monitored individuals. React.js was selected for its robust capability to handle dynamic updates and efficiently render complex interfaces, which are crucial for displaying live data streams from IoT sensors. This dashboard displays real-time data and alerts, enabling caregivers to promptly detect and address critical situations, thereby enhancing their response capabilities. Data management and routing are handled through Node.js [34] and Express.js [38], which efficiently manage data transmission between the IoT sensors and the dashboard. This setup ensures that data handling is both secure and robust, maintaining system integrity and responsiveness under various operational loads.

We prioritized user accessibility in the design of the dashboard to ensure that it remains intuitive and easy to navigate. This approach allows users of all technical skill levels to utilize the dashboard to make informed decisions quickly and effectively.

**Figure 3:**
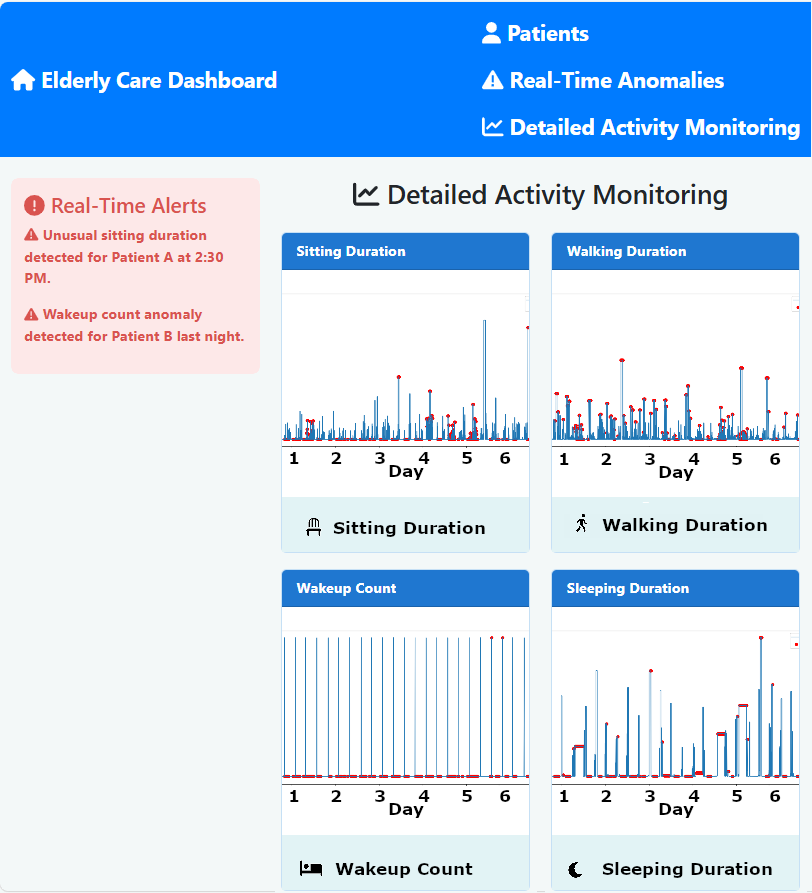
Real-time monitoring dashboard.

Figure 3 illustrates the developed interface of the monitoring dashboard, showcasing its capability to display live data and real-time alerts. The dashboard is designed not only to display health data but also to enable caregivers to immediately react to anomalies and emergencies, thus supporting proactive patient care. The architecture supports dynamic updates and real-time interactions without performance degradation, ensuring scalability. Prior to deployment, the system undergoes rigorous integration testing to confirm that all components function cohesively. This testing includes functional verification to ensure that each module operates correctly and interacts appropriately with other system parts, as well as performance assessments under simulated peak and normal conditions. These developments underscore the crucial role of the dashboard in our system, providing a reliable and effective tool for real-time health monitoring and emergency management in elderly care settings.

## 5. Experiments

This section rigorously examines the experiments conducted to test the implemented components of the IoT system designed to support elderly individuals and their caregivers through edge computing. The experiments demonstrate the system’s real-time operational capabilities, its efficacy in anomaly detection, and the functionality of the user interface, which aids caregivers in making informed decisions.

### 5.1. Detailed Experimental Setup

Our experimental setup utilizes the advanced Raspberry Pi 4 Model B [44], an embedded low-cost computer for prototype experiments [45], as an edge device. It was selected for its robust processing power, which is essential for real-time data analytics and critical for immediate patient care responses at the edge. This edge computing device, equipped with a Quad-core Cortex-A72 (ARM v8) SoC at 1.5 GHz, 4 GB of RAM, and a 32 GB MicroSD card for data storage, manages extensive data processing on-site, minimizing latency crucial for timely interventions. The actual experimental data used in our study were sourced from the TM029 dataset provided by the CASAS project. This dataset includes comprehensive sensor data previously collected in real environments designed to monitor elderly individuals. The data encompass activities like cooking, eating, and sleeping, along with sensor activations such as motion detection, door usage, and temperature readings. To simulate a real-time operational environment, we employed a script that mimicked the presence of real devices. This script retrieves data from the dataset and sends it to our system for processing. The TM029 dataset was collected using non-wearable sensors, allowing us to test our system’s data processing and analysis capabilities under realistic conditions. This approach ensures that our experimental validation reflects the practical application of the technology in real-world settings without the necessity for the physical deployment of sensors during the initial testing phase.

#### 5.1.1. Data Selection and Utilization

The TM029 dataset from the CASAS [12] project, designed to monitor elderly individuals, provided comprehensive sensor data for this study. The dataset includes labeled activities, such as cooking, eating, and sleeping, along with raw sensor activations, such as motion detection, door usage, and temperature readings. These labels enable the system to differentiate between normal and anomalous behaviors.

We conducted a series of preprocessing steps to prepare the dataset for analysis. First, data cleaning was performed using custom scripts in Python to remove outliers and erroneous entries, ensuring data integrity. Subsequently, all numerical features were scaled using min-max normalization, implemented via the Scikit-Learn library, to maintain uniformity across data features. Lastly, timestamp alignment was achieved using the pandas library, which converted all time data into a consistent datetime format, facilitating accurate temporal analysis. This preprocessing ensured that the dataset was ready for feature extraction and machine learning model training.

#### 5.1.2. Feature Engineering for Anomaly Detection

Strategic feature engineering is crucial for enhancing the anomaly detection capabilities of our system, mainly because it fine-tunes the input data for optimal relevance and significance, directly influencing model performance. We developed critical features that track and analyze daily routines and any deviations therein, which are vital for understanding patient behavior and potential health issues. For instance, sitting duration is calculated by aggregating periods when the subject is stationary, which helps identify sedentary behavior patterns critical in elderly care. Walking duration is monitored through motion sensors, providing a quantitative measure of physical activity essential for assessing mobility levels. Similarly, wake-up count is a crucial feature derived from movement data collected during typical sleeping hours, serving as a crucial indicator of sleep quality and disturbances. Out-of-home duration is another significant metric, tracked through door sensor activations to gauge the time spent outside the home, reflecting the individual’s social activity or potential isolation. Lastly, sleep duration is estimated from periods of inactivity during night hours, providing critical insights into the subject’s sleep patterns and overall rest quality. These features were specifically chosen for their strong predictive value regarding health concerns, such as cognitive decline, physical inactivity, and sleep disorders, which are prevalent issues in elderly care. To ensure the effectiveness of these features, each one undergoes a rigorous normalization process to maintain uniformity across different types of measurements before integration into our anomaly detection models. The models use these standardized features to establish behavioral baselines and identify deviations that may indicate potential health problems. This meticulous feature engineering process enables our anomaly detection models to respond effectively and accurately reflect real-world conditions. This capability significantly improves the system’s utility in detecting and addressing emergent health issues, thereby enhancing the overall efficacy of patient care management.

#### 5.1.3. Model Performance Evaluation

Following the extensive preparation and feature engineering detailed in the previous subsections, we rigorously evaluated the performance of our anomaly detection models using a set of standardized metrics crucial for confirming their effectiveness and reliability. Accuracy, which measures the proportion of correctly identified anomalies, provides an overarching view of the model’s correctness. High accuracy indicates that our system is reliable and can be trusted to make critical decisions in real-time applications [46]. Precision is the ratio of true positive anomalies to all positive predictions, highlighting the model’s ability to avoid false positives. Precision is particularly important in elderly care, where unnecessary alerts can lead to alarm fatigue among caregivers. Recall (sensitivity) reflects the model’s ability to identify actual anomalies, emphasizing its detection capabilities. Ensuring high recall is crucial for the safety and well-being of elderly individuals, as missed detections could have serious consequences. The F1 score, the harmonic mean of precision and recall, offers a balanced measure of the model’s performance and is particularly valuable when dealing with imbalanced data [47] typical of anomaly detection scenarios in smart homes. This ensures that both precision and recall are reasonably high, confirming the balanced sensitivity and specificity of the model. By rigorously applying these metrics, we fine-tune the models to optimize performance and reliability, ensuring that the system operates effectively under the diverse conditions found in elderly care environments. This rigorous application of standardized evaluation metrics helps adjust our models to meet operational requirements for real-time anomaly detection, ultimately enhancing the overall efficacy of patient care management.

## 6. Results and Discussion

This section elaborates on the findings from the deployment of our system, which leverages ML models such as isolation forest and LSTM to form a robust framework for supporting elderly care settings. These findings illustrate how the system’s capabilities can significantly impact elderly care through the early warning of potential health issues to doctors and caregivers. As discussed in the previous section, we aimed to demonstrate the system’s feasibility and effectiveness. Scripts were utilized to feed the system real data simulating input from IoT environmental devices. Therefore, the different components of the system acted as expected: they preprocessed the data, applied ML anomaly detection techniques to predict anomalies in the received data, transmitted the results to the cloud block, and displayed them on the dashboard. This section showcases and discusses the different aspects involved in the process.

### 6.1. Integrative Cognitive Health Assessment

The dashboard is a crucial component of our system. It synthesizes data from sleep quality, motor function, and social interaction analyses to comprehensively assess cognitive health. By integrating and analyzing trends and patterns across these key health domains, the dashboard offers a multifaceted solution that enhances the accuracy and timeliness of detecting potential cognitive decline. Therefore, it forms the basis for evaluating the effectiveness and reliability of our system. The specific results displayed on the dashboard, which can help doctors and caregivers in their daily activities, are detailed below. Then, in the subsequent sections, we present the results in terms of performance and accuracy, obtained from the different components that operated in the background to generate each result shown on the dashboard.

**Sleep Quality Analysis**: Sleep quality is a vital indicator of an individual’s overall health and cognitive function. Our system uses motion sensors, as used for the CASAS TM029 dataset [12], to monitor various sleep parameters, such as duration, frequency of awakenings, and signs of restlessness. These sensors passively track movements during sleep to detect disruptions and quantify sleep quality without requiring active participation from the individual. The LSTM model analyzes these data to identify deviations from established sleep norms, which are indicative of potential sleep disorders or broader health issues that may impact cognitive function. This analysis helps caregivers customize interventions to improve sleep quality and, by extension, cognitive health.

**Figure 4:**
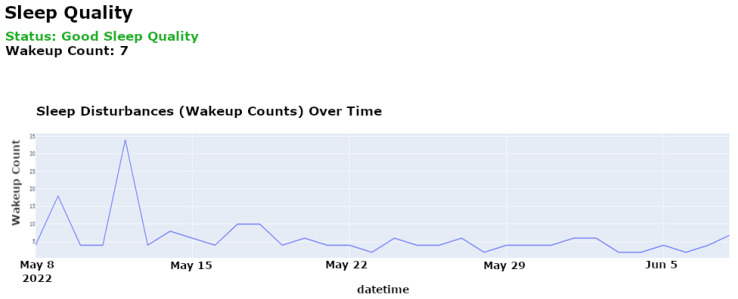
Sleep disturbances.

Figure 4 illustrates specific instances of sleep disturbances over a selected period, highlighting patterns such as frequent awakenings or unusually short sleep durations, which may suggest underlying health conditions like insomnia.

**Figure 5:**
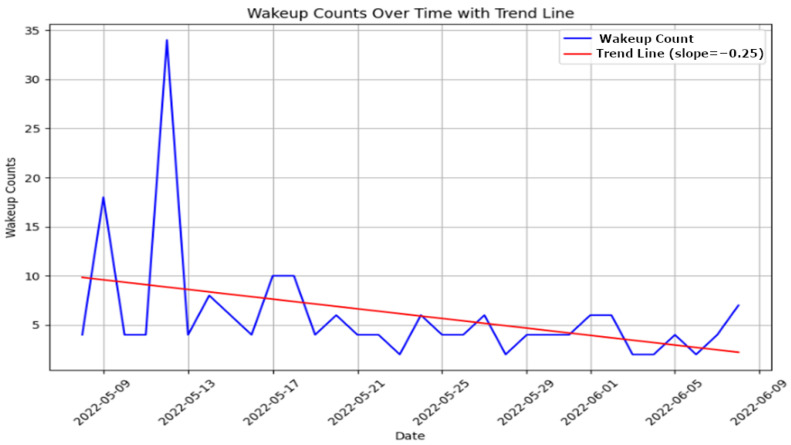
Sleep disturbance trend line.

Figure 5 shows the evolution of sleep quality over time, visualizing how sleep patterns respond to interventions or changes in health status, helping caregivers assess the effectiveness of treatments or identify deteriorating conditions.

**Motor Function Monitoring**: Motor function provides insights into physical capabilities and potential cognitive issues. Motor function monitoring in our system is based on motion sensors and door sensors, as used in the CASAS TM029 dataset [12]. Motion sensors detect walking activity and periods of inactivity, while door sensors track entry and exit patterns. By analyzing the ratio of time spent sitting to walking using data collected from motion sensors, our isolation forest model identifies anomalies in these patterns that may indicate declining mobility, fatigue, or potential cognitive issues. Unusual increases in sedentariness or reductions in mobility may signal physical conditions affecting cognitive status, prompting further assessment and targeted interventions to enhance mobility and cognitive engagement.

**Figure 6:**
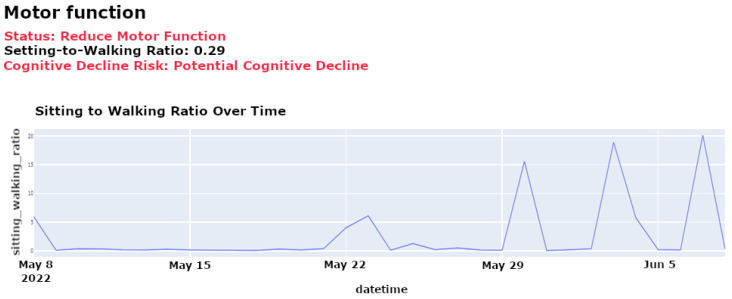
Motor function.

Figure 6 depicts daily ratios of sitting to walking, clearly showing periods of reduced mobility or increased inactivity, which may indicate potential health risks or the need for targeted intervention.

**Figure 7:**
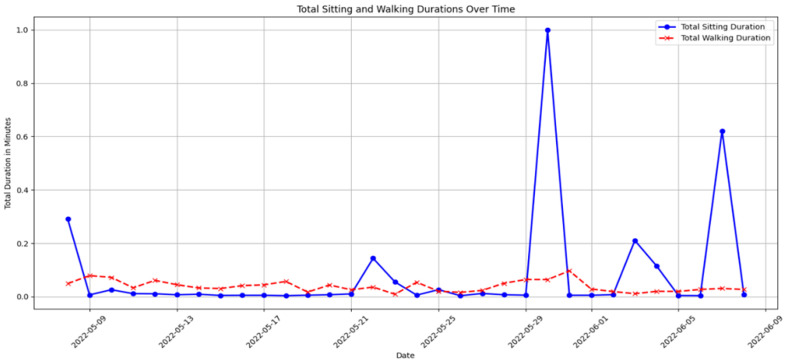
Sitting and walking trend line.

Figure 7 provides a longitudinal analysis of motor function, highlighting trends that may require medical attention or lifestyle adjustments, such as increasing physical therapy sessions or adjusting daily routines to enhance mobility.

**Social Interactions Evaluation**: This feature tracks the frequency and quality of an individual’s interactions with other people both within and outside their living environment. By analyzing variations in engagement patterns, the system detects potential social withdrawal, which may be an early indicator of cognitive or emotional decline.

**Figure 8:**
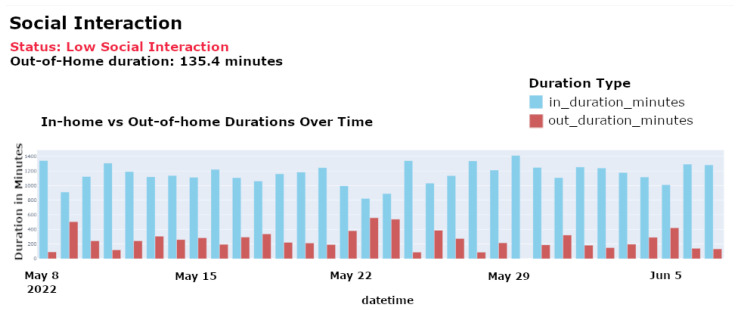
Visualization of social interaction frequency, highlighting periods of reduced engagement that may indicate cognitive or emotional changes.

Figure 8 illustrates the frequency of social interactions over time, identifying periods of reduced engagement that may signal a need for intervention.

**Figure 9:**
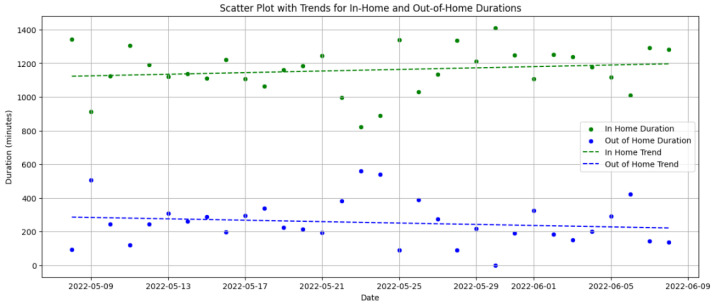
Comparison of in-home and out-of-home social activities, emphasizing behavioral shifts that may suggest social withdrawal or early cognitive decline.

Figure 9 compares home versus out-of-home activities, highlighting any significant changes in social behavior or potential withdrawal from usual activities, which are essential for assessing social habits and detecting early signs of depression that may indicate reduced social participation. Identifying these trends is crucial for initiating timely social stimulation interventions, which support cognitive well-being and mental health.

**Practical Benefits of Anomaly Detection**

Our anomaly detection system supports caregivers and healthcare workers by closely monitoring key health indicators, such as sleep quality, motor function, and social interactions. By identifying anomalies in these areas, caregivers can take timely action to prevent health deterioration and improve elderly care outcomes. For example, our system detected decreased nocturnal mobility and increased awakenings in an 82-year-old male showing signs of cognitive decline. This triggered a telemedicine consultation, where caregivers adjusted his sleep environment and medication, leading to marked improvements in sleep quality and cognitive function. Similarly, the system flagged a decline in mobility in an 85-year-old female, prompting early intervention with physical therapy, which prevented further deterioration. Additionally, our system detected social withdrawal in a 78-year-old male based on reduced social interactions, allowing caregivers to introduce targeted social engagement activities, thereby improving the patient’s overall well-being. These real-world scenarios demonstrate the system’s effectiveness in transforming reactive healthcare into a proactive model, enabling caregivers to respond before serious health conditions develop.

**Comprehensive Cognitive Health Dashboard**

Our dashboard synthesizes data from the aforementioned analyses to provide an integrative view of cognitive health. By correlating sleep quality, motor function, and social interaction data, the dashboard identifies patterns that might not be apparent from individual measurements, ensuring a holistic approach to cognitive health assessment. The described dashboard provides a visual summary of the key metrics it monitors. Based on the aggregated data from the monitored metrics, it illustrates an individual’s overall health status, with specific emphasis on the risk of cognitive decline.

### 6.2. Machine Learning: Anomaly Detection and Model Performance

**Figure 10:**
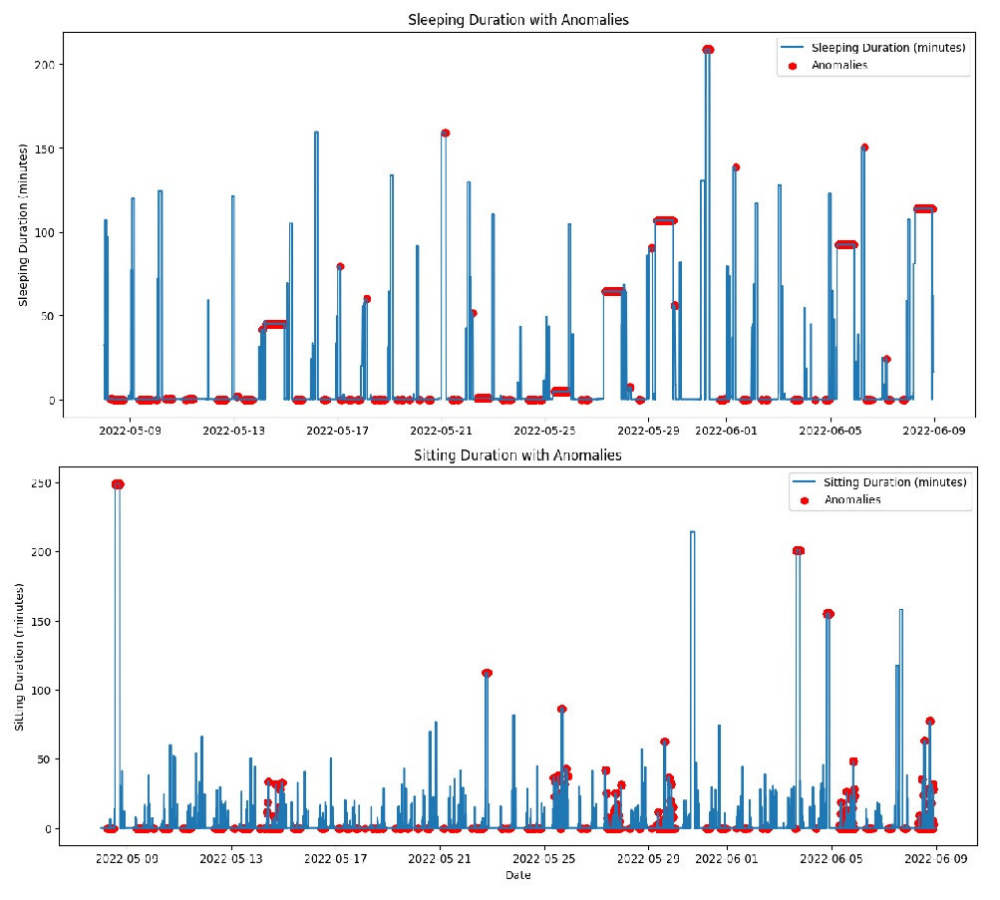
Detected anomalies, part 1.

**Figure 11:**
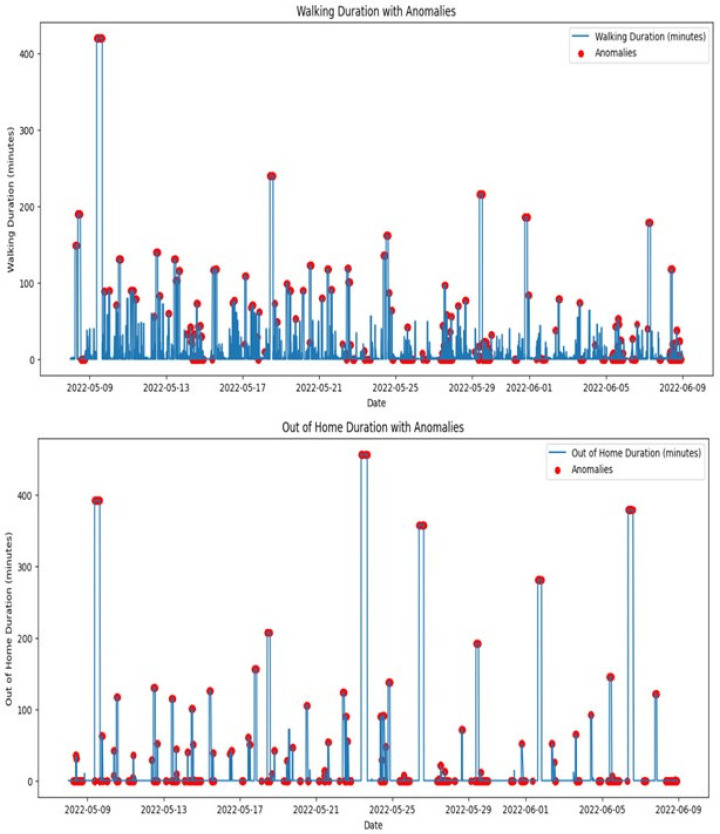
Detected anomalies, part 2.

As discussed, the proposed system employs advanced machine learning models, including isolation forest and LSTM, to effectively detect and analyze anomalies. The performance of these models is critically evaluated by their ability to identify deviations from normal behavior patterns, which are essential for preemptive healthcare interventions. Continuous tuning of the model parameters based on real-time data enhances detection accuracy, particularly in distinguishing between usual behavioral variability and genuine anomalies that require intervention. A critical demonstration of the efficacy of the dashboard was observed when it promptly issued an alert for an unusual sitting duration noted in an elderly patient. This alert enabled caregivers to quickly assess the situation and intervene appropriately, thereby showcasing the ability of the system to facilitate rapid response strategies and contribute to proactive patient management. The isolation forest model is used to identify anomalies in the daily activity patterns of elderly individuals. It effectively detects deviations from normal patterns by applying unsupervised learning techniques. This model isolates anomalies by randomly partitioning the dataset and typically requires fewer splits to isolate anomalous observations from normal instances, which are more abundant and thus deeper in the tree structure. The evaluation of our proposed edge-based anomaly detection system involved analyzing the daily activities of a single resident in a smart home using the CASAS [12] dataset. The key results obtained are shown in Figure 10 and Figure 11.

As previously discussed, the system processes sensor data in real time, ensuring that anomalies are promptly detected and reported. This real-time processing capability is critical for ensuring timely intervention, especially in the context of elderly care. As shown in the previous subsection, machine learning algorithms were applied to various activities, including sleep duration, sitting duration, walking duration, and out-of-home duration, over a one-month period. The results highlight the system’s ability to detect anomalies across these activities. The top graph in Figure 10 shows the daily sleep duration, with red markers indicating anomalies. These anomalies represent deviations from the typical sleep pattern. Days with significantly reduced sleep durations could indicate interrupted sleep or early waking times, which may be symptoms of sleep disorders or disturbances in the living environment. Consistent anomalies in sleep duration require attention as they could indicate underlying health issues. It is essential for caregivers or medical professionals to investigate these irregularities further to provide appropriate interventions and ensure the well-being of elderly people. Therefore, our system is able to generate real-time notifications based on identified irregularities or critical events, ensuring that caregivers and healthcare providers are promptly informed of potential issues. The bottom graph displays the daily sitting duration, with red markers indicating anomalies, i.e., prolonged periods of inactivity. Such patterns could be concerning, particularly for elderly individuals at risk of health complications associated with sedentary behavior. Extended periods of sitting without breaks can contribute to several health issues, including cardiovascular disease and increased risk of falls due to muscle weakening. Addressing these anomalies with scheduled activities or reminders to move could improve health outcomes.

The top graph in Figure 11 shows the daily walking duration, with red markers indicating anomalies. Deviations from the norm could suggest mobility issues or irregular daily activities. For instance, days with low values could point to reduced mobility. Monitoring walking duration is crucial for assessing elderly adults’ physical activity levels and mobility. Anomalies might prompt assessments such as physical therapy evaluations or adjustments in daily mobility aids. The bottom graph illustrates out-of-home durations, with anomalies indicated by red markers. Deviations in these patterns could signify unexpected absences or changes in the usual routines of the individual, such as not leaving the house at all on days they normally would or being away for unusually long periods. Anomalies in out-of-home duration could raise safety concerns or highlight changes in social activities. The LSTM (Long Short-Term Memory) model was employed to analyze temporal dependencies within the activity data, enhancing the detection and understanding of anomalies in the behavior of elderly individuals. The model’s capability to process time-series data makes it particularly well suited for monitoring systems in smart homes, where activities and their patterns over time are crucial for accurate anomaly detection.

**Figure 12:**
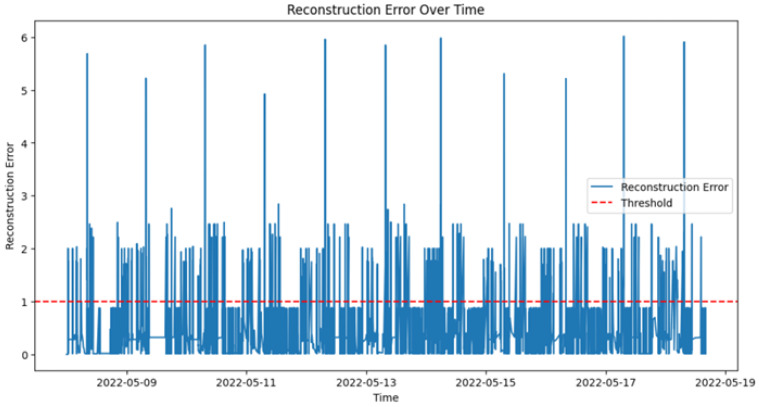
Reconstruction error over time.

Figure 12 shows the reconstruction error over time for the Long Short-Term Memory (LSTM) network, which is used to detect anomalies in activity data. The reconstruction error is calculated as the difference between the predicted and actual values. In the figure, significant spikes in the reconstruction error indicate instances where the observed activity deviated from the expected normal behavior, leading to anomaly detection. The threshold for anomaly detection was set at a reconstruction error of 0.25, determined through cross-validation to minimize false positives while ensuring sensitivity to potential health risks. Several instances exceeded this threshold during the observation period, with the highest reconstruction error reaching 0.35, signaling unusual behavior that warranted further investigation. Early stopping was applied during the training process after five epochs of no improvement in validation loss, ensuring that the model generalized effectively to new data.

**Figure 13:**
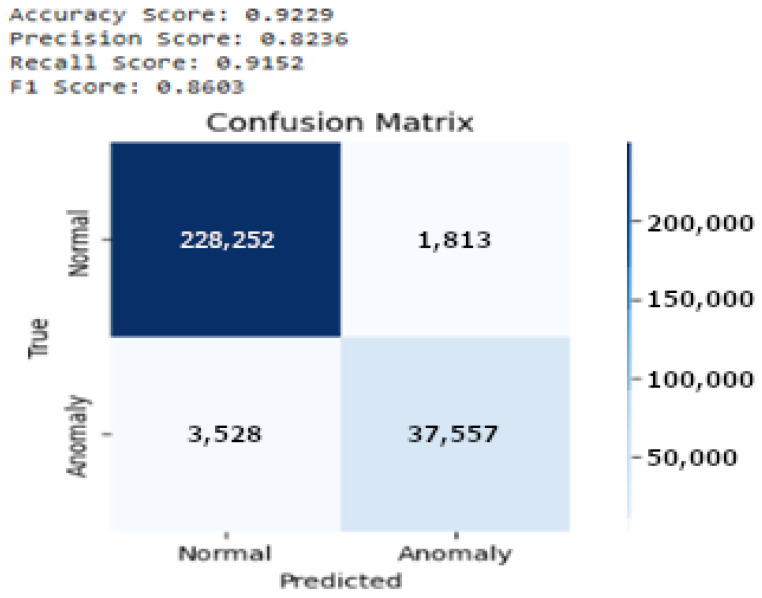
Confusion matrix and performance metrics.

The performance of the proposed model is shown in Figure 13, detailed through a confusion matrix, demonstrating strong predictive accuracy, with 37,557 true positives and 228,252 true negatives, reflecting the effectiveness of the model in accurately identifying both anomalies and normal behavior. It also records 1813 false positives and 3528 false negatives, indicating some areas for improvement. The model achieved an overall accuracy of 92.29%, a precision of 82.36%, a recall of 91.5%, and an F1 score of 86.6%. These metrics highlight the capability of the model to reliably detect most anomalies with a high degree of precision; however, the presence of false negatives suggests the potential for further refinement. Enhancements could include more nuanced feature engineering, training dataset expansion, or anomaly classification threshold adjustments. Continuous model evaluation and updates are crucial to adapt to evolving patterns of normal and anomalous behavior, ensuring that the model remains effective over time.

**Figure 14:**
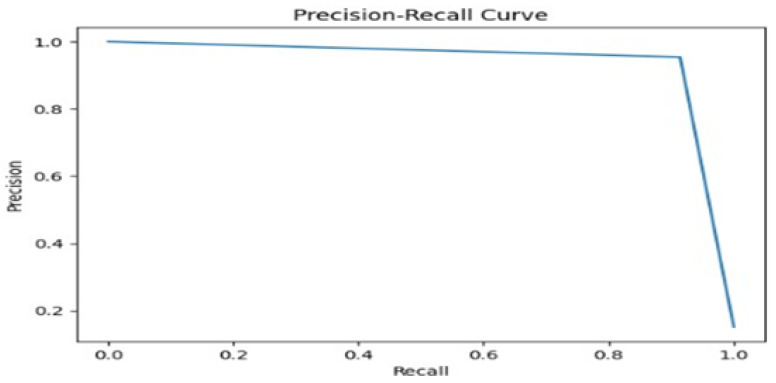
Precision–recall curve.

Further, in Figure 14, which presents the precision–recall curve, we can observe that the curve begins with a high precision close to 1, indicating that the model was highly accurate in predicting anomalies, especially at lower levels of recall. As recall increases, precision declines sharply, suggesting that the model started to compromise accuracy for a broader coverage of potential anomalies.

**Figure 15:**
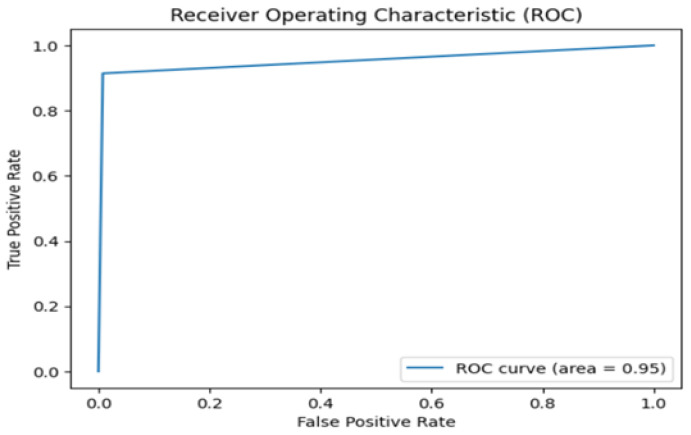
Receiver Operating Characteristic (ROC) curve.

Figure 15 presents the ROC curve for the LSTM model used in anomaly detection. The curve plots the true positive rate (TPR) against the false positive rate (FPR) at various classification thresholds. The Area Under the Curve (AUC) is 0.95, indicating that the model effectively distinguished between normal and anomalous behavior. The steep rise of the ROC curve in the early stages reflects a high TPR with a minimal increase in the FPR, emphasizing the ability of the model to accurately detect anomalies with minimal false alarms, which is crucial in real-time monitoring systems for elderly care.

Table 2 comprehensively compares the different anomaly detection methods used in related works. It highlights the accuracy, precision, recall, and F1 score of various models and methods employed across different studies, along with the datasets used. Each study’s real-time context, key technologies, and targeted anomalies are detailed, offering a clear perspective on how our approach stands in comparison. This table underlines our study’s unique approach in employing a combination of isolation forest and LSTM models to handle multi-activity anomaly detection, leveraging the CASAS TM029 [12] dataset to enhance real-time monitoring capabilities in smart homes.

### 6.3. Dashboard: Integration and Utility Monitoring

#### 6.3.1. Heatmap of Activities (Normalized)

**Figure 16:**
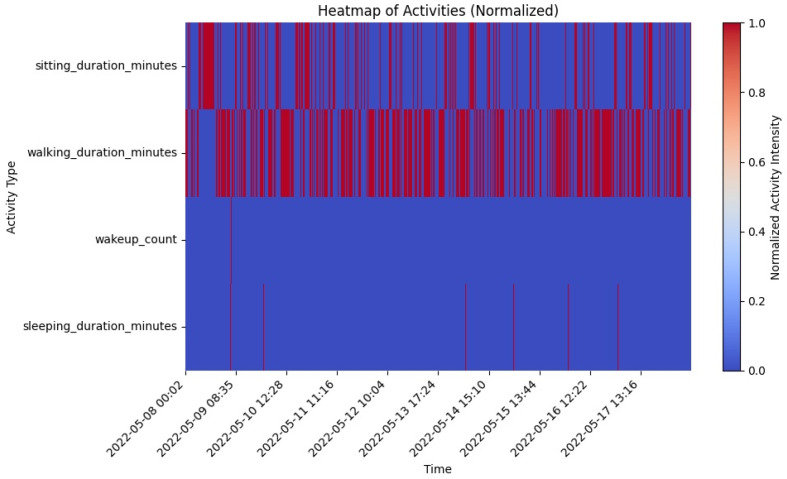
Heatmap of activities.

The heatmap presented in Figure 16 illustrates the normalized intensity of four key activities, i.e., sitting duration, walking duration, wake-up count, and sleep duration over 10 days. The activities were normalized using min-max scaling, where the color scale ranges from blue (low intensity) to red (high intensity). This visualization allows for a comparative analysis of the intensity and frequency of each activity, making it easier to identify trends, shifts, or potential anomalies in daily behavior. This heatmap is integrated into the monitoring dashboard, enhancing its utility by providing a clear, color-coded overview of activity patterns that help caregivers make informed decisions. The insights gained from the heatmap analysis directly inform care strategies, enabling dynamic adjustments to care plans to better accommodate the day-to-day variability in the individual’s activities and conditions.

The x-axis represents the timeline, while the y-axis displays the four monitored activities. This graphic representation helps quickly identify periods of higher or lower activity levels and offers insight into the daily routines of elderly adults. The walking duration activity shows the highest overall intensity, as evidenced by the prevalence of red bands throughout the 10 days, suggesting that the individual frequently moved throughout the day, with regular fluctuations in activity levels. However, the heatmap also reveals sharp drops in walking activity, represented by the blue sections, which could indicate potential health concerns, such as mobility issues or fatigue. These deviations may be flagged by the anomaly detection models, particularly the LSTM model, as potential areas of concern. The sitting activity displays a more sporadic pattern, alternating high- and low-intensity periods. Red bands that indicate prolonged sitting suggest periods of reduced movement, which may be normal during specific times of day but could also signal potential risks, such as prolonged inactivity. The isolation forest model may flag longer sitting durations as potential anomalies, particularly if they coincide with a reduction in walking. Wake-up events are less frequent and are represented by sporadic red spikes, and they are generally expected to occur once a day. However, multiple wake-up events or an increased frequency of wake-up events, such as nighttime disruptions, might indicate sleep disturbances. Anomalies in wake-up patterns may indicate underlying health issues such as insomnia or restlessness, and the isolation forest model may highlight these. Sleeping patterns appeared relatively stable across the 10-day period, with consistent blue bands representing the times when the individual slept. Any significant reductions or fragmentations in sleeping patterns, visible as breaks in the blue sections, could signal potential sleep disorders or disturbances. These sleep irregularities could be captured by the LSTM model, providing early warnings of deteriorating sleep quality.

#### 6.3.2. Time-Series Plots of Activity Patterns

**Figure 17:**
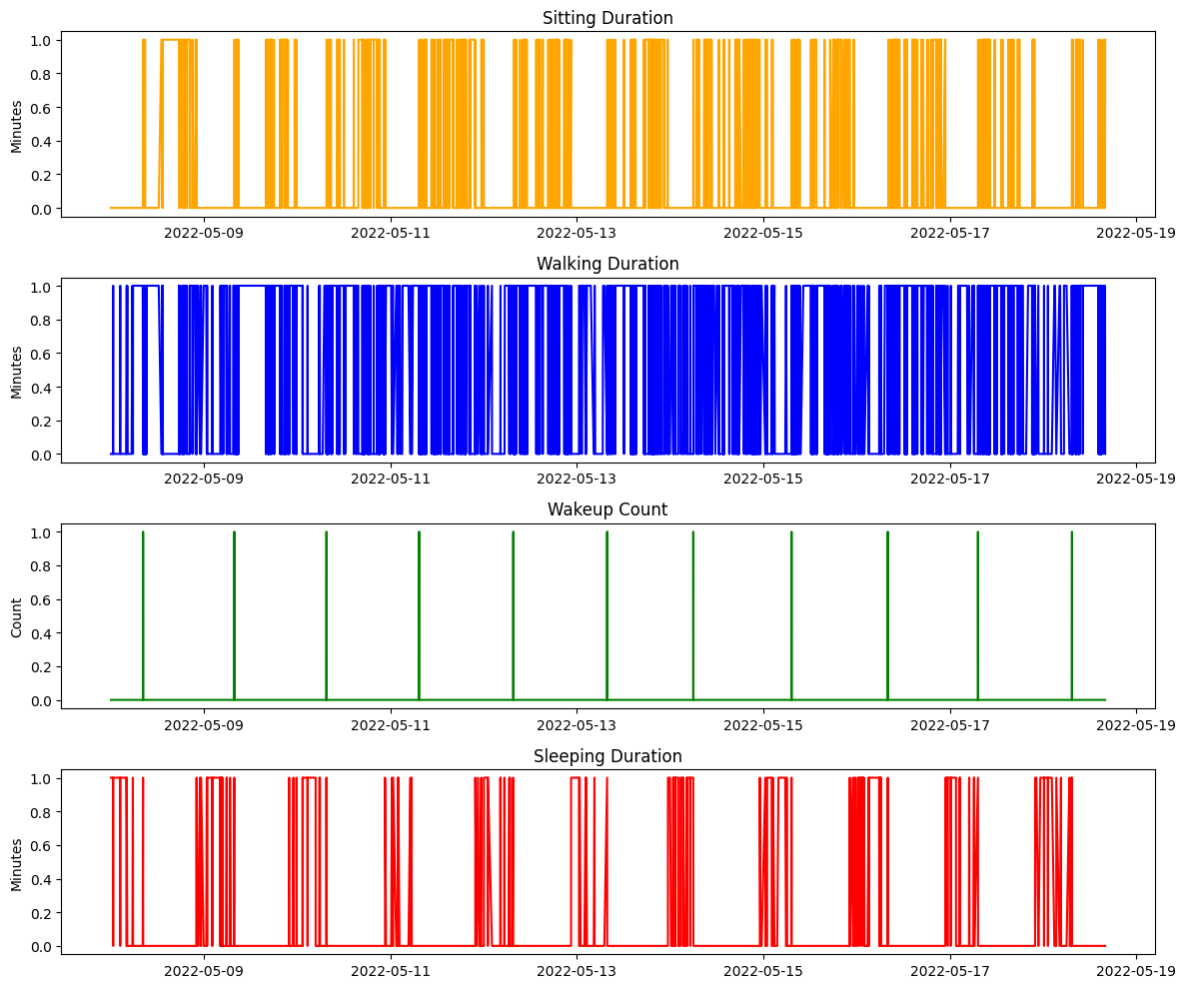
Time-series plots displaying the daily activity patterns over the 10-day period.

The time-series plots presented in Figure 17 illustrate the daily activity patterns of four key activities: sitting duration, walking duration, wake-up count, and sleep duration over a 10-day observation period. These visualizations provide critical insights into temporal fluctuations in activity levels and allow for the identification of potential deviations from typical behavior. The monitoring dashboard, as shown in Figure 3, complements these plots by providing real-time visual feedback on these activities, allowing caregivers to promptly respond to detected anomalies directly from these interfaces. The x-axis represents the timeline across 10 days, while the y-axis depicts the normalized durations for sitting, walking, and sleeping or the count of wake-up events. Each activity is represented by a distinct color: sitting duration is shown in orange, walking duration in blue, wake-up count in green, and sleep duration in red, enabling a clear distinction between activities and facilitating the detection of trends or anomalies.

The time-series plot of the sitting duration reveals the frequency and length of time spent sitting over the 10-day period. The data show frequent short intervals of sitting interspersed with occasional extended periods. High variability was observed in the duration of sitting periods, suggesting normal day-to-day fluctuations in the daily routine. However, prolonged sitting periods may indicate potential health concerns, such as fatigue or reduced mobility, and can be classified as anomalies. The isolation forest model successfully flagged several instances where extended sitting durations deviated from the norm. These instances were flagged on the monitoring dashboard (as shown in Figure 3), highlighting them as anomalies for caregiver intervention. The walking duration plot shows regular movement throughout the day, indicating periods of mobility. The relatively consistent walking patterns suggest stable mobility, with only a few instances of reduced walking duration. However, significant drops in walking could signal mobility issues or health concerns. For example, on certain days, noticeable reductions in walking were flagged by the LSTM model as anomalies. These deviations could indicate potential fatigue or difficulties in movement, which are crucial to elderly care monitoring. These deviations were prominently displayed on the monitoring dashboard, allowing caregivers to quickly identify and address these mobility issues. The plot shows wake-up events as distinct spikes, representing transitions from sleep to wakefulness. Typically, one or two wake-up events were expected each day, corresponding to the normal sleep cycle of the individual. However, an increased number of wake-up events within a day may indicate disturbed sleep patterns, potentially signaling sleep disorders or other health-related concerns. The isolation forest model flagged days with increased wake-up counts as potential anomalies, drawing attention to possible sleep disturbances. The sleep duration plot shows regular sleep periods during the 10-day observation window, with most sleep occurring at night. Consistency in sleep patterns is a positive indicator of stable health. However, deviations from typical sleep patterns were observed on certain days, including shorter sleep durations or frequent interruptions, which could indicate sleep-related health concerns such as insomnia. The LSTM model successfully identified these deviations, flagging them as anomalies for further investigation. Additionally, these visualizations provided a comprehensive overview of the elderly individual’s daily routines, enhancing the capability of caregivers to effectively monitor and respond to anomalies. Enhanced data analysis from the time-series plots has enabled the implementation of targeted interventions, such as mobility exercises and sleep quality improvements, to address the specific needs identified through anomaly detection.

### 6.4. Lessons Learned

Through the development and implementation of our system, several key insights emerged, shaping our understanding of real-world deployment challenges and opportunities for future improvements.

Initially, the integration of ML models, particularly the isolation forest and LSTM models, demonstrated strong potential for identifying behavioral anomalies. By leveraging time-series analysis and anomaly scoring, we were able to flag potential cognitive health concerns early, enabling caregivers to take proactive measures. However, thanks to the modular nature of our system, experimenting with new ML techniques or optimizing the already used ones could improve efficiency in large-scale applications. In addition, we noticed that the variability of home environments, sensor placements, and user behaviors influenced the system’s performance. Future improvements should focus on increasing the diversity of training data to enhance model adaptability and robustness across different real-world settings. Furthermore, despite the stated advantages of edge computing, increasing sensor deployment may increase computational demands. Therefore, any system modification or integration must always consider scalability. Moreover, we demonstrated that using non-wearable sensors can improve compliance, but privacy concerns remain a key focus area that needs to be managed and safeguarded in any future development. Finally, although the experiments demonstrated the system’s efficacy in providing valuable insights to doctors and caregivers, further real-world experiments could expand the dataset to validate the system’s effectiveness in cases of data variability. Additionally, they could motivate the addition of new real-time services to further assist all stakeholders, including patients.

These lessons provide a roadmap for future studies, ensuring that the system continues to evolve as a reliable and scalable solution for elderly care.

## 7. Conclusions

The global population is aging rapidly, with the number of people aged 65 and over expected to double by 2050. This demographic shift presents significant challenges to healthcare systems, emphasizing the need for innovative solutions to ensure the safety and well-being of elderly individuals living independently. Monitoring systems that detect health issues early are essential to enable timely intervention and reduce the strain on caregivers and healthcare resources.

Traditional monitoring approaches rely heavily on cloud computing and wearable devices, which face notable challenges, including high latency, privacy risks, and low compliance among elderly people due to discomfort or forgetfulness. These factors lead to unreliable data collection and hinder real-time decision-making.

In this paper, we present an innovative IoT edge-based framework designed for the remote monitoring of elderly adults that integrates non-wearable sensors, edge computing, and machine learning-based anomaly detection. Our approach leverages anomaly detection techniques to accurately identify behavioral deviations in elderly individuals. Unlike conventional methods, our system processes data locally within the smart home environment, enhancing privacy, reducing latency, and ensuring seamless real-time monitoring. Furthermore, the system offers innovative services, such as a doctor-focused dashboard and a caregiver application, which provide timely alerts and long-term behavioral insights, supporting healthcare professionals and caregivers in proactive decision-making. This design enables seamless, non-intrusive monitoring, eliminating the need for active participation from elderly individuals and effectively addressing the compliance challenges posed by wearable devices.

A prototype of the proposed framework was implemented to evaluate its feasibility and showcase its scalability, efficiency, and reliability in elderly care settings. In our experiments, two of the most frequently used ML anomaly detection models, i.e., isolation forest for spatial anomaly detection and LSTM for temporal analysis, were utilized to identify behavior anomalies in sleep quality, motor function, and social interactions, which are key indicators of cognitive decline.

The findings revealed that the system successfully detected anomalies in daily activities, delivering valuable insights into cognitive and physical health. The experimental evaluation demonstrated that the system achieved 92.2% accuracy, highlighting its effectiveness in early health risk detection and anomaly detection in elderly care.

Future work will include developing the system’s unaddressed components, particularly focusing on enhancing predictive capabilities by integrating advanced machine learning models to improve the early detection of cognitive decline. We plan to utilize more recent relevant studies like the one conducted by Rahman et al. [49], in which a comprehensive review of ML approaches for anomaly detection in smart homes was presented. Additionally, we will expand the dataset to further study the best configuration of ML models to provide the most valuable outcomes to doctors and caregivers. The dashboard and application will also be further extended and adapted to provide suggestions and feedback to elderly individuals, enhancing engagement and usability. Finally, the edge-based nature of the designed system will be leveraged to further test its effectiveness in real scenarios, which will be conducted in collaboration with hospitals and doctors.

Our system has created a basis for developing comprehensive edge-based systems that aim to improve the quality of life of the elderly while also ensuring their safety and well-being through creative, innovative technological solutions.

## Figures and Tables

**Table 1:** Summary of original and derived parameters.

Original Parameter	Derived Parameter	Description
Sensor ID	Sitting Duration	Time spent seated during the day
Timestamp	Walking Duration	Total time spent moving within the home
Motion Sensor Readings	Wake-Up Count	Frequency of awakenings during sleep periods
Door Sensor Activity	Out-of-Home Duration	Time spent outside the home
Environmental Sensors (e.g., temperature)	Sleep Duration	Total sleep time as a measure of rest quality and overall health

**Table 2:** Comparison of different anomaly detection methods used in related works.

Study/Paper	Model/Method	Dataset	Accuracy (%)	Precision (%)	Recall (%)	F1 Score (%)	Real-Time	Context	Technologies	Key Anomalies
Our Paper	Isolation Forest + LSTM	CASAS TM029	92.29	82.36	91.52	86.03	Yes	Smart homes	Python [31], Node.js [34], Express.js [38]	Multi-activity (walking duration, sitting duration, sleep duration, wake-up events)
[14]	LSTM + SMOTE	Anomaly detection for fallen people	84.00	97.00 (normal), 19.00 (anomalous)	60.00 (anomalous)	91.00 (normal), 28.00 (anomalous)	Yes	Elderly care	Python [31], TensorFlow [48]	Fall detection
[13]	Parametric statistical method	Real-world IoT data; 9 participants	Not reported	Not reported	Not reported	Not reported	No	Smart homes	Python [31], custom stats software	Daily activity monitoring
[11]	Graph-based approach	Kyoto (CASAS)	Not reported	Not reported	Not reported	Not reported	No	Smart homes	Python [31], graph tools	Cognitive decline indicators
[15]	SOM + Markov models	MavHome	>75% (Anomaly detection)	Not reported	Not reported	Not reported	Yes	Smart homes	Python [31], SOM, Markov models	Inactivity; unusual presence
[19]	Binary sensors; activity maps	CASAS HH126 and Kasteren	Not reported	Not reported	Not reported	Not reported	No	Smart homes	Python [31]; custom algorithms	Behavioral changes

## Data Availability

The original data presented in the study are openly available in [TM029 dataset from CASAS] at [https://casas.wsu.edu/datasets/].
